# Mimicking the brain: Epstein-Barr virus and foreign agents as drivers of neuroimmune attack in multiple sclerosis

**DOI:** 10.3389/fimmu.2023.1304281

**Published:** 2023-11-03

**Authors:** Olivia G. Thomas, Tomas Olsson

**Affiliations:** ^1^ Therapeutic Immune Design, Centre for Molecular Medicine, Department of Clinical Neuroscience, Karolinska Institute, Stockholm, Sweden; ^2^ Neuroimmunology Unit, Department of Clinical Neuroscience, Centre for Molecular Medicine, Karolinska Institute, Stockholm, Sweden

**Keywords:** autoimmunity, Epstein-Barr virus, T cell, multiple sclerosis, cross-reactivity, antibody, central nervous system, autoreactive B and T cells

## Abstract

T cells have an essential role in adaptive immunity against pathogens and cancer, but failure of thymic tolerance mechanisms can instead lead to escape of T cells with the ability to attack host tissues. Multiple sclerosis (MS) occurs when structures such as myelin and neurons in the central nervous system (CNS) are the target of autoreactive immune responses, resulting in lesions in the brain and spinal cord which cause varied and episodic neurological deficits. A role for autoreactive T cell and antibody responses in MS is likely, and mounting evidence implicates Epstein-Barr virus (EBV) in disease mechanisms. In this review we discuss antigen specificity of T cells involved in development and progression of MS. We examine the current evidence that these T cells can target multiple antigens such as those from pathogens including EBV and briefly describe other mechanisms through which viruses could affect disease. Unravelling the complexity of the autoantigen T cell repertoire is essential for understanding key events in the development and progression of MS, with wider implications for development of future therapies.

## Introduction

MS is the second most common cause of neurological disability amongst young adults after trauma affecting approximately 2.8million people globally (1). MS is more common in females with onset generally between 20 to 40 years of age, and occurs due to the formation of focal inflammatory demyelinating lesions in the CNS. Initial disease has a predominantly inflammatory component and approximately 85% of cases present with a relapsing-remitting phenotype, but over time accumulated damage and neurodegenerative mechanisms can lead to permanent disability. Genetic aetiology is estimated to be around 20-30% for MS with the remaining risk lying with stochastic events and environmental factors such as obesity, smoking, low serum vitamin D and EBV (2).

The brain and spinal cord, once thought to be an immune privileged compartment (3), are surveyed by patrolling T cells which guard against infection and also have roles in brain development and behaviour (4–8). However, the CNS is sensitive to inflammation-mediated damage and therefore, under healthy conditions, a carefully controlled balance is maintained between protection from infections and prevention of injurious inflammation. Loss or impairment of such CNS T cell immunosurveillance leads to an increased risk of CNS infections or malignancies (9).

Focal CNS lesions in early MS disease show widespread inflammatory infiltrates which contain a variety of immune cells including CD4^+^ T cells, CD8^+^ T cells B cells and monocytes. Genome-wide association studies (GWAS), as well as clinical therapeutic observations, indicate that T cells and B cells alongside innate immune cells have a key roles in MS neuroinflammatory mechanisms (10–15). The majority of MS-associated genes have functions in antigen presentation, cytokine production, proliferation, T helper (T_H_) cell differentiation, co-stimulation, signal transduction and function. Associated polymorphisms have been identified in the human leukocyte antigen (HLA) locus as well as in *IL-2Rα*, *IL-7Rα*, *CXCR5*, *CD40*, *CD86*, *STAT3* and many other genes (15–17). However, the strongest known genetic risk factor associated with MS is the HLA-DRB1*15:01 allele with an odds ratio (OR) of approximately 3 (15–18), and has also been shown to interact with multiple other environmental risk factors to increase risk further (19–24). The HLA locus also contains several other class II alleles which confer risk for developing MS and several identified HLA class I alleles which protect from disease such as HLA-A*02:01 (17, 25). CD4^+^ T cells recognise peptides presented in the context of HLA class II molecules, whereas CD8^+^ T cells recognise class I-presented peptides. Given that the function of HLA is to present peptides to T cells for recognition via T cell receptors (TCR), the association of HLA-DRB1*15:01 with MS development suggests a role for class II-presented peptides and autoreactive CD4^+^ T cell responses and has led to substantial investigation of the autoantigens responsible for priming of pathogenic responses.

Early research established in particular the role of CD4^+^ T cells which target these antigens due to several observations, such as the presence of CNS-infiltrating CD4^+^ T cells in MS brain lesions, genetic risk conferred by *HLA-DR* and *HLA-DQ* alleles, increased experimental autoimmune encephalomyelitis (EAE) susceptibility of transgenic mice expressing MS-associated HLA class II molecules, and it is likely that CD4^+^ T cells also contribute to MS pathogenesis via their influence on both adaptive and innate immune processes such as antibody production by B cells and CD8^+^ T cell maturation.

To prevent production of T cells which can target self-tissues and trigger autoimmunity, T cells undergo central tolerance mechanisms in the thymus during their maturation, where cells expressing T cell receptors (TCRs) that bind strongly to peptide:HLA complexes on thymic epithelial cells and dendritic cells are negatively selected, and those that do not bind at all die by neglect (26). Additional mechanisms in the periphery also act to remove self-reactive T cells which escape central tolerance (27). Due to these mechanisms, TCRs which pass thymic tolerance quality control and bind foreign antigens generally have high affinity for their cognate antigen, however some responses may also have the ability to bind other peptides presented on HLA at different affinity which could include those from self-antigens. The classical view of T cells is that a single TCR expressed at the cell surface allows them to bind one peptide:HLA complex but the reality is that a single TCR can likely bind multiple peptides, and possibly also different HLA molecules (28). This existence of cross-reactivity might be an evolutionary advantage of a limited genome-encoded TCR repertoire against the myriad of possible peptide combinations which can occur in nature, estimated to be around 10^15^ possible peptides (29, 30). This T cell degeneracy indicates a potential for TCRs which were originally selected during exposure to prior foreign antigens to also bind self-peptides presented by HLA, and therefore the pathogens which we encounter throughout life shape both the memory T cell repertoire as a whole and also its autoreactive potential.

As well as T cells, there is strong evidence supporting a role for B cells in MS development, in particular due to the dramatic therapeutic effect of anti-CD20 therapies (11, 12). The reasons for which include B cell antigen presentation to T cells, production of pro-inflammatory cytokines, elimination of Epstein-Barr virus (EBV), and finally production of pathogenic autoantibodies – not in prime focus for this review. This review will discuss the current knowledge surrounding T cell specificity in MS, evaluating the existing evidence that CNS autoreactive responses may originally have been generated in response to non-self antigens and briefly describing other mechanisms through which viruses could affect disease.

## Selected evidence for the role of T cells in MS

For many years CD4^+^ T cells have been considered an important cell type involved in MS pathogenesis due to early observations in experimental autoimmune encephalomyelitis (EAE) – the animal model for MS – that CNS demyelinating disease can be transferred by adoptive transfer of myelin-reactive CD4^+^ T cells (31, 32), and further evidenced by the observation that EAE cannot be transferred by antibodies alone. The role of CD4^+^ T cells in MS has been further demonstrated by the strongest genetic susceptibility conferred by HLA class II alleles (15), susceptibility of HLA class II-carrying mice to demyelinating disease (33–36), presence of CD4^+^ T cells in inflammatory brain lesions (37), and the involvement of CD4^+^ T cells in several other arms of adaptive immunity such as antibody production and CD8^+^ T cell maturation.

T_H_1 and T_H_17 CD4^+^ T cells have been linked to MS disease with identification of these specific subsets in MS brain lesions and correlation of T_H_1 cytokine-producing cells in peripheral blood with MS relapses (38–41). Further studies have also demonstrated a role for a unique intermediate population of T_H_1-like T_H_17 CD4^+^ T cells in MS which are associated with relapse, predominant in the CSF of early disease pwMS and can be isolated from MS brain lesions (42, 43). High avidity CD4^+^ T cells with specificity for selected myelin antigens have also been shown to produce interferon-γ (IFNγ) and have a T_H_1 phenotype in persons with MS (pwMS) (44–47). A clinical trial of a myelin basic protein (MBP) altered peptide ligand (APL) showed exacerbations in some MS patients, and further investigation showed cross-recognition between the APL and MBP driven by CD4^+^ T cells which were skewed towards a T_H_1 phenotype (45). In addition to T_H_1, CD4^+^ T cells with a T_H_17 phenotype have also been detected in MS brain lesions and have been shown to be necessary for the development of EAE (48, 49). Follicular helper CD4^+^ T cells (T_FH_) cells provide help to B cells for their maturation, affinity maturation and antibody production, and germinal centre formation, and this essential link between humoral and cellular immunity makes them of key interest in MS pathology due to the involvement of B cells in disease. Activated T_FH_ cells have been shown to be increased in peripheral blood, their frequency correlated with disability and are detectable in brain lesions in MS (50–52).

The role of CD8^+^ T cells is less clear though several observations suggest their involvement in MS such as high abundance in MS lesions, low or transient expression of HLA class I molecules on the surface of microglia, oligodendrocytes and neurons (53, 54), and observations that EAE does not develop in B2-microglobulin knockout mice (55). Several HLA class I associations with MS have also been identified, such as the strongest known protective alleles HLA-A*02:01 and HLA-B*38:01 ( 25) mentioned above, although untangling HLA associations with disease is notoriously difficult due to linkage disequilibrium and also other factors such as killer-immunoglobulin receptor (KIR) type, which can have a significant effect on immune activation in natural killer (NK) cell subsets (56). In addition, HLA-A*02:01 protection against MS may be related to actions in the type I interferon system rather than peptide binding and activation of CD8^+^ T cells (57). Myelin-reactive CD8+ T cells have also been characterised – although to a lesser extent than CD4^+^ T cells – and have been isolated from both pwMS and healthy individuals. Studies of brain-infiltrating CD8^+^ T cells in MS have shown their TCR repertoire to be oligoclonal, suggesting antigen-specific migration or expansion within the CNS (58, 59), and other studies have directly enumerated autoantigen-specific CD8^+^ T cells from peripheral blood of pwMS (60, 61).

## Mechanisms of pathogenic cross-reactivity

A long array of infectious agents have been associated with MS however the strongest evidence lies with EBV and – to some extent – human herpesvirus 6A (HHV-6A) (2, 62–65). In addition to this, the long-list of MS-associated autoantigens has grown in recent years which has broadened the focus of research in this area and led to some debate on which antigens and pathogens are pathologically relevant in neuroimmunological demyelinating disease (66–72). The association of these viruses and other pathogens in the context of the molecular mimicry with CNS autoantigens in MS will be discussed in this review.

**Figure 1:**
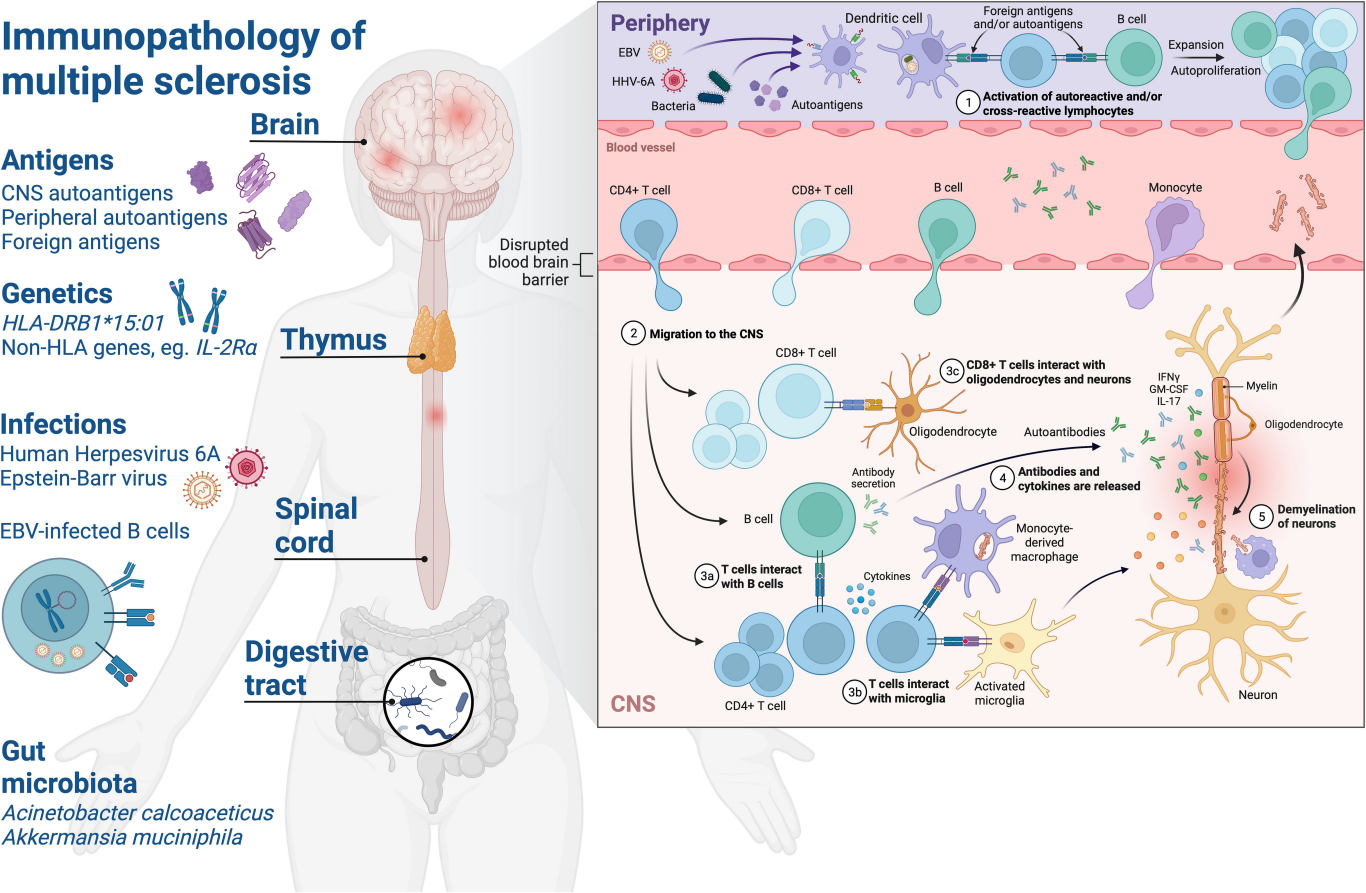
Schematic of the immunopathology of multiple sclerosis. Environmental and genetic factors affect development of neuroinflammatory mechanisms in MS. Exposure to foreign antigens primes humoral and cellular immune responses with cross-reactivity to self-antigens in the CNS. Human leukocyte antigen (HLA) – HLA-DRB1*15:01 – on the surface of APC present foreign or autoantigen peptides and shape the CD4^+^ T cell repertoire via central tolerance mechanisms in the thymus. Infections such as EBV and HHV-6A are associated with increased MS risk and drive elevated antibody responses to viral proteins which have been observed in pwMS, such as increased EBNA1-specific antibodies. The role of the EBV-infected memory B cell compartment in MS is not fully understood, but may be attributed to several factors which could predispose to autoimmunity such as: providing a source of molecular mimicry epitopes throughout life which can prime pathogenic and cross-reactive CD4^+^ T cell and antibody responses, processing and presentation of antigens to prime T cells, production of proinflammatory cytokines, rescuing of autoreactive B cells from apoptosis and/or modulation of innate and adaptive immune mechanisms via expression of viral immune evasion proteins. Gut microbiota associated with MS such as Acinetobacter calcoaceticus and Akkermansia muciniphila may influence CNS autoimmunity by providing molecular mimicry epitopes or driving a proinflammatory milieu which could lead to breakdown of immune tolerance via bystander activation of autoreactive T and B cells. (1) Priming of T cells and B cells with reactivity to foreign antigens and/or autoantigens in the periphery, autoproliferation is driven via interactions between T cells and B cells in pwMS and leads to clonal expansion. Elevation of antibodies with reactivity to viral and/or autoantigen in the periphery. (2) Blood-brain barrier alterations lead to migration of lymphocytes to the CNS where they mediate inflammation. (3) CD4^+^ T cells interact with B cells and activated microglia in the CNS, CD8^+^ T cells interact with neurons and oligodendrocytes via HLA class I molecules. (3) Antibodies and cytokines are released driving inflammation and lesion formation. (4) Breakdown of myelin sheaths leads to neuronal axonal damage and demyelination over time. CNS, central nervous system; HLA, human leucocyte antigen; EBV, Epstein-Barr virus; HHV-6A, Human herpesvirus 6A; IFNγ, interferon-γ; IL-2Rα, interleukin-2 receptor α; IL-17, interleukin-17; GM-CSF, granulocyte macrophage stimulating factor. Created with BioRender.com.

MS has long been associated with previous EBV infection (73) and, while the exact mechanism remains to be fully characterised, the different theories have been summarised previously (65) (**Figure 1**). Despite uncertainty surrounding the sequence of events which eventually lead to MS, it has been established that EBV infection almost always precedes disease development. There is in fact a delay between infection and onset of neurological symptoms and also a lack of neurological symptoms in individuals with acute symptomatic EBV infection, also known as infectious mononucleosis (IM) (73–76). This interval between infection and neurological disease onset could potentially reflect a time delay between initial priming of pathogenic immune responses and epitope spreading within antigens or to new ones, which over time leads to inflammatory demyelinating disease. In support of this view, altered adaptive immune responses to EBV antigens have been identified in MS (77–80) and some have been found to cross-react with human proteins, leading researchers to conclude that molecular mimicry may have a key role in MS development.

Despite many links between viruses and MS, direct evidence that viruses can trigger molecular mimicry leading to initial CNS autoimmune demyelinating disease is lacking in humans. However, proof of concept was shown in an EAE model using recombinant Theiler’s murine encephalomyelitis virus (TMEV) expressing a naturally occurring proteolipid protein (PLP) molecular mimic from *H. influenzae*. Early onset EAE could be induced in SJL/J mice by TMEV infection, but disease was not triggered when the same peptide was used to vaccinate in complete Freud’s adjuvant, suggesting that virus activation of antigen presenting cells (APC) is necessary for disease development in this model (81). An additional study by Ji et al. used a recombinant Vaccinia virus expressing myelin basic protein (MBP) to trigger autoimmunity in Rag2^-/-^ mice expressing an MBP-specific CD8^+^ TCR (82). Disease was also triggered in mice after infection with the wild type Vaccinia vector which did not contain MBP but immunisation with peptide plus adjuvant only did not trigger disease, indicating that viral infection was necessary to break tolerance to CNS antigens in these models. A further study investigating the role of CD8^+^ T cells used a mouse model where oligodendrocytes expressed ovalbumin (OVA) and showed that even high numbers of high avidity OVA-specific CD8^+^ T cells could not induce EAE, and that these cells were in fact deleted from the immune repertoire to prevent autoimmunity under normal non-infected CNS conditions or during peripheral infection with OVA-expressing Listeria bacteria. In contrast, when mice were intracerebrally infected, OVA-specific CD8^+^ T cells destroyed oligodendrocytes and induced demyelination (83).

These examples from EAE indicate that autoreactive T cell responses to myelin antigens are not solely sufficient to initiate CNS autoimmunity and that additional triggers are required to break tolerance, such as virus-mediated activation of APC or a blood-brain barrier permeabilisation event. In support of this view is the observation that myelin-specific T cells can be detected in healthy individuals (84, 85), however it could be that activation of these autoreactive T cells in the periphery through molecular mimicry to foreign antigens skews them towards a pathogenic phenotype capable of migrating to and targeting CNS tissue (**Figure 1**).

## Particular examples of cross-reactivity between CNS and foreign antigens in MS

A general problem with most descriptions of molecular mimicry T cell specificities is to know if these indeed have pathogenic roles or whether they represent innocuous epiphenomena, which is difficult to prove in humans. However, for some, there is epidemiological evidence for an association to disease and in the following paragraphs we discuss a set of autoantigen mimicry suspects. A full list of the foreign antigen cross-reactivity with CNS proteins discussed in this article is summarised in **Table 1**.

**Table 1:** Selected molecular mimicry between foreign and host CNS antigens in MS.

Autoantigen	Human T cell response	Human B cell/antibody response	EAE	T cell molecular mimicry	B cell/antibody molecular mimicry
Myelin oligodendrocyte glycoprotein (MOG)	(45, 85–92)	(87, 93–97)	(98–103)	Human NFM (104)	Butyrophilin (105, 106), HERV-W (*in vitro* only) (107), Influenza-A virus haemagglutinin (108), *Acinetobacter* sp. 3-oxo-adipate-CoA-transferase subunit A (EAE) (93)
Proteolipid protein (PLP)	(45, 47, 109–113)	(94, 113–116)	(117, 118)	Human coronavirus 229E (119), Human coronavirus OC43 (119), *A. castellanii* (EAE model) (120), *H. influenzae* (EAE model) (81, 121), *S.cerevisae* (CD8+) (122)	-
Myelin Basic protein (MBP)	(45, 46, 123–129)	(93, 94, 97, 116, 128, 130, 131)	(36, 102, 103, 132–134), HBV polymerase molecular mimicry (135)	EBV BALF5 (136–138), Herpes simplex UL15 (136), Herpes simplex DNA polymerase (136), Adenovirus type 12 ORF (136), *Pseudomonas* sp. phosphomannomutase (136), HPV type 7 L2 (136), Influenza type A HA (136), Reovirus type 3 sigma 2 protein (136), EBV EBNA1 (EAE) (139), EBV LMP1 (EAE) (140), HHV-6 U24 (141), Human coronavirus 229E (119, 142), Human coronavirus OC43 (119), human and bacterial GDPLFS (70), Vaccina virus (EAE) (82), large T antigen JC virus (EAE) (143), Herpesvirus saimiri (EAE) (144), Cpn0483 *C. pneumoniae* (EAE, rat MBP) (145).	EBV EBNA1 (139, 146), EBV LMP1 (140, 147), *Acinetobacter* sp. 4-CMLD (EAE) (93), *P. aeruginosa* γ-CMLD (93)
α-crystallin B (CRYAB)	(69, 148)	(69, 94, 97, 149, 150)	Priming in EAE (69) (103, 151),	–	EBV EBNA1 (69, 152)
Glial cell adhesion molecule (GlialCAM)	(153)	(153)	(153)	–	EBV EBNA1 (153)
Anoctamin-2 (ANO2)	–	(67, 68)	-	–	EBV EBNA1 (68)
RAS guanyl releasing protein 2 (RASGRP2)	(71, 72)	-	-	HLA-DR (71), EBV BPLF1 (71), EBV BHRF1 (71), *A. muciniphila (* 71), HLA-DR-derived self-peptides (71)	-
GDP-L-fucose synthase (GDPLFS)	(70, 92)	-	-	Bacterial GDPLFS (70), MBP (70)	-
HLA-DR-derived self-peptides	(71)	–	–	RASGRP2 (71), EBV BHRF1 (71), EBV BPLF1 (71), *A. muciniphila (* 71)	–
Myelin-associated glycoprotein (MAG)	-	(94, 97, 154)	(102)	–	Bovine casein (154)

## EBNA1 as a source of mimicry epitopes to Anoctamin-2, α-crystallin B and Glial cell adhesion molecule

Several studies have shown that elevated antibody responses to certain antigens from EBV are elevated in MS, in particular immunoglobulin G (IgG) responses to the EBNA1_380-440_ region have a MS odds ratio of approximately 8 and have also been shown to interact with HLA-DRB1*15:01 to increase disease risk further (19, 24, 64). In addition, elevated serum neurofilament levels have been shown to positively correlate with EBNA1 IgG in MS, indicating that there is a relationship between CNS injury and humoral responses to EBNA1 which occurs before disease onset (73). However it is not fully understood what is causing elevated EBNA1 antibody responses in MS and, as this elevation is specific to MS and interacts with HLA-DRB1*15:01, may suggest that molecular mimicry with CNS antigens are driving the EBNA1 antibody response. In support of this view, several epitopes within the MS-associated EBNA1 region have been identified with similar amino acid sequences to CNS autoantigens (68, 69, 139, 153). On the other hand, increased EBNA1 antibody responses in MS may be due to a frustrated EBV-specific immune response, where HLA-DRB1*15:01 is a poor class II allele in the context of EBV immune control. In one study humanised mice that were immune reconstituted from HLA-DRB1*15:01 ^+^ donors had increased steady state activation of CD4^+^ and CD8^+^ T cells and poor virus control evidenced by high EBV viral loads, compared to mice which were reconstituted with an allele not associated with increased MS risk (155). These findings suggests a synergistic interaction between EBV infection and HLA-DRB1*15:01 which primes a hyperactive adaptive immune compartment, leading to poor viral control and facilitating the generation of cross-reactive pathogenic T cell responses.

Α-crystallin B (CRYAB) is a small heat shock protein which is expressed in oligodendrocytes and has been shown to have paradoxical roles in MS: both in protection from harmful inflammatory innate immune mechanisms via chaperone activity and also conversely as the target of adaptive T cell responses in a proinflammatory environment (148–150, 156). This multifaceted role somewhat confounded the MS field and cast doubt on CRYAB’s role as an autoantigen in MS, however antibody responses to CRYAB were recently revisited in a large Swedish cohort and showed 27.6% of pwMS and 16.9% of controls to have IgG responses to CRYAB peptides with homology to EBNA1, and were associated with MS (OR=1.98) (69). Risk was further increased with an OR of 8.99 when combined with high EBNA1 IgG responses in individuals. Reciprocal blocking experiments showed that CRYAB IgG responses were blocked when the homologous EBNA1 epitope was spiked into sera, and the core homologous epitope between these antigens was mapped to a RRPFF motif at CRYAB_11-15_ and EBNA1_402-406_. Similarly, another group identified cross-reactive antibodies targeting the RRPFF motif in oligoclonal bands of pwMS (152), and interestingly the CRYAB sequence contains a PxxP motif similar to that found in MBP and several antigens from herpesviruses discussed later in this review. In addition, the high frequencies of EBNA1- and CRYAB-specific T cells observed in natalizumab-treated pwMS produced IFNγ and immunisation of mice with CRYAB or EBNA1 protein elicited T cell responses to the reciprocal antigen, also indicating cross-reactivity on the T cell level (69). The role of CRYAB as an autoantigen is complex and autoantibodies such as those that target intracellular antigens may not be directly pathogenic, however generation of high affinity antibodies depends on T cell help, and therefore they could be markers of a T cell response which is able to target intracellular antigens in autoimmune disease. In support of this view, B cells have been shown process and present epitopes from the antigen that they have Ig specificity with greater efficiency to T cells which have TCRs that respond to the same antigen (157). In addition, LMP1 expression has been shown to enhance antigen presentation and co-stimulation in EBV-transformed B cells via CD70, OX40 ligand and 4-1BB, which may have a role in priming of pathogenic autoreactive T cell responses (158).

The presence of CRYAB IgG in only a subset of pwMS suggests involvement of other autoantigens in the non-responders, and a recent study by Lanz et al. identified clonally expanded plasmablasts in the CNS of pwMS and identified their target epitope as a sequence shared between EBNA1 and glial cell adhesion molecule (GlialCAM) (153). These antibodies bound a core epitope within the MS-associated region of EBNA1 at residues 394-399 which is directly next to the CRYAB homologous epitope and cross-reacted with GlialCAM_377-383_, again both sequences contain PxxP motifs. In addition to this, the authors noted that affinity for the GlialCAM epitope was increased with the phosphorylation of the serine residue at position 376, and increased antibody reactivity to these core epitopes was also demonstrated in the plasma of pwMS compared to controls. SJL/J mice which were initially immunised with EBNA1_386-405_ peptide had a worse EAE disease course compared to a scrambled peptide control and the mice developed antibody responses to GlialCAM indicating generation of cross-reactive responses. Also similar to CRYAB, GlialCAM is expressed by oligodendrocytes and astrocytes in the CNS and is also present in chronic active lesions of MS (159, 160). Whilst no study so far has investigated both GlialCAM and CRYAB responses in individuals, it would be interesting to understand whether pwMS have responses to both antigens or whether these are restricted by different HLA. It is possible that the close proximity of these epitopes could lead them to be differentially processed and presented on surface HLA, or it could lead to their restriction by the same HLA.

Anoctamin-2 (ANO2) is a calcium-activated chloride channel with 8 membrane-spanning domains which is predominantly expressed in neurons and glial cells in the CNS, and also has high expression in the retina and in MS lesions (67). ANO2 was identified as a target of autoantibodies in MS in a large screening study and responses were found to be positively associated with HLA-DRB1*15:01, with an adjusted OR of 17.3 (67). Furthermore, combination of several risk factors including ANO2 IgG positivity, high EBNA1 IgG, presence of HLA-DRB1*15:01 and absence of HLA-A*02:01, produced a combined OR of over 26 (68). A later study went on to confirm the association of ANO2 IgG with MS in a larger cohort of almost 16,000 individuals, and identified cross-reactivity of ANO2_140-149_ antibody responses with EBNA1_431-440_ which is also in the MS-associated region (68). In addition to HLA-DRB1*15:01, 14 other HLA alleles were found to be associated with ANO2 IgG levels, indicating that epitopes from ANO2 can be presented by multiple different HLA and providing indirect evidence that there may also be ANO2 T cells in MS. Interestingly the strongest HLA effect on ANO2 IgG levels was a protective effect for HLA-DRB1*04:01, which the authors speculate could be due to increased elimination of high affinity ANO2-specific T cells in the thymus; the same effect was also observed for EBNA1 IgG levels, again indicating potential cross-reactivity on the T cell level.

Given that EBNA1-specific antibodies have now been reported in several studies to cross-react with multiple human proteins including MBP (discussed later in this review), CRYAB, GlialCAM and ANO2 (68, 69, 139, 146, 153), all of which were found to be elevated in pwMS and were associated with disease, what evidence is that EBV infection may have triggered these responses? Tengvall et al. showed that ANO2 IgG responses could be detected in a pre-MS cohort (68), indicating that responses appear before clinical onset of disease, an observation that has also been published for EBNA1 IgG (73). Additionally, it is very rare to detect antibody responses to ANO2, GlialCAM and CRYAB in individuals without evidence for a prior EBV infection, particularly EBNA1 IgG, indicating that EBV infection may be a prerequisite for the development of these autoantibodies and implying that molecular mimicry may be driving development of these responses. Presence of a CRYAB IgG response was also shown to be negatively correlated with ANO2 IgG in individuals, suggesting that these autoantibodies likely do not develop in the same individuals which could be due to factors such as HLA type (69) and that different autoantigens lead to MS disease in individuals.

However, the autoantibodies with cross-reactivity to EBNA1 described above were each only detected in a relatively small subset of pwMS, suggesting that these cross-reactive antibodies are not necessary for disease in all patients. It is likely that further undiscovered autoantigen cross-reactivity exists or it could be that the cross-reactive antibodies are not themselves pathogenic in the majority of MS cases, but are instead biomarkers for a T cell response which is responsible for mediating autoimmune damage, as has been observed for other autoimmune diseases such as Addison’s disease and diabetes mellitus (161, 162). Additionally, GlialCAM and ANO2 epitopes are both within intracellular domains and CRYAB is expressed intracellularly and are therefore not exposed, making it difficult to assume direct autoantibody-mediated damage to the CNS; although this cannot necessarily be excluded as several examples exist of pathogenic autoantibodies which bind intracellular targets such as GAD65, proinsulin and IA-2 in diabetes (163). Plasmapheresis is only therapeutically effective in a subset of MS patients (164) which suggests that it is not the antibodies themselves that are responsible for MS disease but the pathogenic T cell responses that they mark. Although positive responses to plasmapheresis in patients with histological lesion patterns type I and II, and particularly in individuals who showed signs of a humoral response, could indicate that autoantibodies drive disease in some individuals (165). In addition to this, evidence for T cell responses to EBNA1 mimics has been shown both in EAE immunisation models and in humans (69, 139, 153) and, although no direct evidence for cross-reactivity on the single T cell level has been so far shown, this is likely to be present, although the potential relevance of these responses to MS development and progression remains to be determined.

Other scant reports of further antibody cross-reactivity between EBV proteins and autoantigens have been described such as that of EBNA1 IgG with heterogeneousnuclear ribonucleoprotein L (HNRNPL) (166), although these were not found to be increased in the plasma of pwMS compared to controls. Antibody cross-reactivity has also been reported for BFRF3 with septin-9 and BRRF2 with the mitochondrial protein dihydrolipoyllysine-residue succinyltransferase (DLST), although again these responses were only in a subset of patients and need to be confirmed in larger cohorts (167). As with most of these reports, validation in large cohorts are required to identify the frequency of these responses in patients and their relevance to neuroinflammatory disease, however the mounting reports of cross-reactivity between EBV and self-antigens suggest that there are multiple disease relevant autoantigens in MS and that each individual may have specific profiles of reactivity.

Another potential consideration is the promotion of tolerance breakdown and molecular mimicry by EBV-mediated immune dysregulation. EBV encodes several viral mimics of human proteins with essential roles in immunity and have evolved over thousands of years to facilitate viral escape from the immune system, and one such example is the viral CD40 mimic latent membrane protein 1 (LMP1). CD40 is a co-stimulatory molecule expressed on APC and is particularly essential for B cell activation via its function as a co-receptor for the B cell receptor (BCR), interaction with CD40 ligand (CD40L) on T cells and amplification of innate mechanisms such as TLR signalling (168–172). LMP1 has been demonstrated to self-aggregate and facilitate downstream signalling in B cells with promote activation, germinal centre formation and production of cytokines and antibodies (173). One study of a transgenic mouse model which constitutively expressed the cytoplasmic tail of LMP1 showed that animals were prone to autoantibody production and immune dysregulation but had no signs of clinical disease (174). However, when these animals were immunised with EBNA1, they showed markedly increased inflammatory cellular and humoral responses compared to animals without LMP1 with T cells producing IFNγ and IL-17 cytokines. Additionally, expression of LMP1 was shown to drive molecular mimicry between EBNA1 and the systemic lupus erythematosus-associated autoantigen Sm (174). Incidentally, the Sm-homologous epitope is at EBNA1_398-404_ and overlaps almost identically with the region reported to contain homology to GlialCAM and CRYAB in MS (69, 153). Whilst the authors do not report increased neurological symptoms in these animals, it would be pertinent to also investigate whether transgenic expression of LMP1 also facilitates molecular mimicry with CNS autoantigens.

Other EBV-encoded mimics of host antigens include BCRF1 which contains homology to interleukin-10 (IL-10) and BHRF1 which is a mimic of Bcl-2, proteins which limit host immune responses to pathogens and promote survival of infected B cells respectively (175, 176). In addition to this, multiple EBV proteins have been shown to modulate antigen processing and presentation in infected B cells, suggesting even further ways in which the virus may shape adaptive immune responses to mimicry epitopes (176–179), and this is an avenue in which there has been very little research in the context of autoimmunity. Given these observations, one can easily imagine how high expression of EBV-encoded immune mimics and modulators such as LMP1 during acute infection or IM may facilitate the breakdown of tolerance, and indeed history of IM has been demonstrated to increase MS risk (19, 22, 23). Of further relevance to MS immunopathology is how EBV-mediated modulation of immunity differs throughout life from childhood to adolescence given the increased risk of developing MS with delayed EBV primary infection (62). For example, studies in mice have shown that a specific population of early-differentiated natural killer (NK) cells expand prior to CD8+ T cells and is involved in virus control during early infection (180, 181). Further study of the analogous population in humans showed that early differentiated NK cells diminished with age and may be involved in protection of children from EBV infection and IM (182). Furthermore, proliferative and cytokine production of CD56^BRIGHT^ NK cell have been demonstrated to be diminished in pwMS (183) indicating that this population may also be impaired in its response against EBV, but further study of NK cell function in pwMS are needed to establish their relevance.

It is evident that cross-reactivity occurs between CNS and viral antigens, however the time and space of exposure to virus antigens may be one of the key determinants for developing MS. Indeed, does EBV’s unique life cycle and persistence in the B cell compartment throughout life mean that this virus is uniquely positioned to trigger CNS autoimmunity? Evidence for this could be derived from observations that the T cell and antibody response to EBNA1 only emerge 3-6 months post primary infection (184) and the delay also exists between seroconversion and development of MS in individuals (74).

## Myelin basic protein

Proof of concept that virus peptides could induce CNS autoimmunity to was shown by Fujinami and Oldstone in 1985, where rabbits immunised with either MBP or a homologous peptide from Hepatitis B virus (HBV) polymerase developed EAE (135). Since then, multiple studies in both human and animal models have demonstrated the ability of T cells generated against MBP to target other antigens.

T cell molecular mimicry between myelin and EBV antigens in humans was first reported by Wucherpfenning and Strominger who isolated T cell clones which responded to MBP_85-99_ (136). Amongst the epitopes which activated the MBP_85-99_-specific T cell clones was a peptide from EBV DNA polymerase (BALF5_627-641_) in the context of MS-associated alleles HLA-DRB1*15:01 and HLA-DRB5*01:01 respectively, and these clones were subsequently tracked to the cerebrospinal fluid (CSF) of patients and the TCR:peptide-HLA structure solved (137, 138). This cross-recognition of BALF5 and MBP peptides in the context of different HLA molecules demonstrated that TCRs can even recognise different complexes as long as there is similar overall structure and charge of residues. Interestingly, the same study showed that MBP_85-99_-specific T cell clones were also activated by multiple other viral and bacterial epitopes – some of them with no clear amino acid homology to the original MBP peptide – including peptides from human papilloma virus (HPV), herpes simplex virus and influenza A virus amongst others (136).

The long-established association of elevated EBNA1 IgG with MS suggests that this response may have a role in disease pathology. Early research by Bray et al. discovered two homologous epitopes in EBNA1 and MBP and isolated antibodies with specificity for the MBP-homologous EBNA1 epitope from oligoclonal bands in the cerebrospinal fluid (CSF) of 85% pwMS in their cohort (146). This provided early evidence that, rather than simply biomarkers, elevated EBNA1-specific IgG responses may target myelin and have a role in MS disease mechanisms. A later study identified antibody responses to EBNA1_411-426_ which were specific to untreated MS-patients and were also able to bind MBP_205-224_ (139). Whilst the study cohort was small, IgG responses to EBNA1_411-426_ were higher in untreated pwMS but low/no responses could be detected in individuals undergoing interferon-β therapy. Furthermore, mice immunised with EBNA1_411-426_ amounted both T cell and antibody responses to MBP, despite low amino acid sequence homology between these two regions (139). Whilst this data from a mouse model is intriguing and suggests T cell cross-reactivity, there are currently no examples of dual-reactive human EBNA1-specific T cells which have been investigated on the single cell level. It is also important to note that oligoclonal bands in MS have been shown to contain specificities for multiple viruses in addition to EBV EBNA1, and therefore there is some debate around their role in CNS autoimmunity (185), however EBNA1 remains a top candidate for molecular mimicry.

Sequence similarity between MBP and the EBV latent antigen LMP1 was also identified by a small study which used phage display to recreate clones from B cells in peripheral blood of pwMS (147). Reverse engineering of sequences from MBP-specific antibody variable domains showed similarity to previously identified LMP1-specific antibody sequences, and were subsequently found to bind recombinant LMP1 by Western blot. Further *in vivo* analysis showed that MBP- or LMP1-immunised mice produced antibody responses to the reciprocal antigen, and comparison of responding B cell repertoire clonality was suggestive of greater epitope spreading in the LMP1-immunised animals (140). CD4^+^ T cells from LMP1-immunised mice also showed proliferation following *in vitro* MBP re-stimulation suggesting cross-priming of T cell responses *in vivo* (140). Despite these findings, no obvious amino acid homology exists between MBP and LMP1 antigens, however this does not necessarily exclude the presence of structurally similar epitope/s as has been previously shown (186). However, as for EBNA1 T cell responses, comprehensive analysis of LMP1-specific adaptive responses in MS cohorts is needed to determine the relevance of this cross-reactivity for disease pathogenesis.

Epidemiological evidence has also linked HHV-6A to increased risk for developing MS, and antibody responses to immediate early protein 1 (IE1) from HHV-6A in a pre-MS cohort showed an increased MS risk with an OR of 2.22 (63). Interestingly, this effect was only observed for IE1 IgG responses to HHV-6A and not for the HHV-6B strain which was instead negatively associated with disease (OR=0.74) (63). As the study used sequence variation in IE1 to distinguish between infection with HHV-6A and 6B, it is so far not known whether the risk associated with HHV-6A IE1 IgG responses is due to the IE1 response itself or the virus that it marks. However, a previous study by Tejada-Simon et al. identified dual-specific T cell responses with reactivity to MBP_96-102_ epitope and HHV-6 U24_4-10_ which share 6 out of 6 identical amino acids across a PxxP motif (141). Approximately 50% of T cells which responded to MBP also responded to this HHV-6 U24 epitope and produced predominantly T_H_1 cytokines and patients with dual-specific responses also had increased antibody titres to both peptides, indicating a direct link between dual-specific cellular and humoral responses to the same epitopes (141). HHV-6A and B strains share over 90% sequence identity and the U24 PxxP amino acid motif which is relevant for cross-recognition with MBP is conserved between both A and B strains. However, different phosphorylation patterns in the MBP-homologous U24 region may affect immune recognition of this epitope or interaction with cellular proteins (187). Alternatively, the difference in MS risk between these strains could be due to increased susceptibility to HHV-6A infection of KIR2DL2-carrying MS patients or to U24-mediated disruption of MBP-Fyn interactions which stabilise myelin (188, 189), and further research is needed to elucidate the exact mechanisms of HHV-6A in MS.

Proof that a virus peptide with homology to a myelin antigen with a core of only 5 amino acids could induce disease in EAE was first demonstrated in by Gautam et al. in 1998, where a PxxP motif peptide from Herpesvirus Saimiri with amino acid homology to MBP_1-11_ was able to induce disease in EAE (144). PxxP amino acid motifs can be found in MBP and multiple other proteins from herpesviruses such as HHV-6 U24, Human Herpesvirus 7 (HHV-7) U24, human Cytomegalovirus (HCMV) UL25 and UL42, Varicella Zoster Virus (VZV) ORF0, Herpes Simplex Virus-1 (HSV-1) UL56 and EBV LMP2A – the latter of which contains four PxxP motifs (189). These viruses are all in the Herpesviridae family and persist in the human host throughout life, although they have vastly different cell tropisms and immune evasion mechanisms which may affect the availability of antigens to prime responses. As previously mentioned, several of these viruses are associated with MS risk – with EBV and HHV-6A showing increased odds ratios (62, 63, 190) whilst CMV is associated with protection (191, 192). So far, T cell molecular mimicry has only been identified between HHV-6 U24 and MBP in humans, and it is possible that sequence of infection with these viruses throughout childhood and early adulthood shapes the T cell repertoire, predisposing some individuals to CNS autoimmunity through cross-reactivity. Further research is needed to establish the relevance of cross-reactive MBP PxxP motifs with viral antigens, and in particular how this might develop throughout challenge with multiple homologous epitopes from viruses.

It seems evident that multiple virus infections have the potential to induce immune responses which cross-recognise MBP, however this may be in part due to pre-existing T cells in the periphery with low to moderate avidity for MBP which escape thymic tolerance mechanisms, despite some expression of MBP in the thymus (193, 194). The escape of MBP-specific T cells from negative selection during central tolerance may be due to the generally lower avidity with which MBP peptides bind to HLA-DRB1*15:01 rendering them unstable, and peptides from MBP have also been reported as promiscuous binders to multiple HLA class II alleles (123, 195, 196). One study demonstrated the ability of an MBP-specific TCR to bind peptide:HLA with a wide range of orientation angles which is possibly due to the scarcity of hydrogen bonds between at the TCR:peptide interface, and this low affinity interaction may explain TCR degeneracy and escape from thymic negative selection (197). MBP-specific T cells can also be detected in healthy individuals which suggests that they occur naturally but are less frequent, have a less pathogenic phenotype, do not gain access to the CNS or are prevented from causing disease by regulatory or other mechanisms under normal conditions (46, 124, 198–202). Differences in MBP peptide immunodominance have also been identified between pwMS and controls (40, 124) although other studies have found no changes in peptides targeted (109, 203, 204). However, it is plausible that environmental exposure to pathogens which contain molecular mimics to MBP – such as to Herpesviruses – could prime or skew responses to different epitopes with higher potential to cause CNS inflammation leading to MS development; so far there is only sero-epidemiological evidence supporting for a role for EBV in MS and to some extent HHV-6A.

More recently, microbiome studies in MS cohorts have led researchers to investigate elevated antibody responses to some bacterial species – such as *Acinetobacter calcoaceticus*, *Akkermansia muciniphila* and *Pseudomonas aeruginosa* – for potential molecular mimicry (205, 206). These studies showed that antibodies from pwMS with reactivity to MBP_43-57_ could bind to epitopes from both *A. calcoaceticus* and *P. aeruginosa* 4- and γ-carboxymuconolactone decarboxylase (CMLD) respectively (93). A similar homology between myelin oligodendrocyte glycoprotein (MOG) and 3-oxo-adipate-CoA-transferase subunit A from *Acinetobacter* species was identified (93), however only the MOG_43-57_-immunised animals showed any sign of disease activity in ABH mice and disease could not be induced by immunisation with the homologous bacterial peptides. On the other hand, molecular mimicry is not the only mechanism through which these bacteria have been suggested to play a role in MS, and studies have shown that *A. calcoaceticus* and *A.muciniphila* species are increased in the gut microbiota of pwMS. Mice which are mono-colonised with these bacterial species have a more severe EAE disease course and produce more proinflammatory adaptive immune responses with fewer IL-10-producing regulatory T cells (T_REG_) (207). In theory, induction of a proinflammatory environment by these bacteria in the MS host could help to skew pre-existing molecular mimicry responses to a pathogenic phenotype which could lead to or influence progression of CNS autoimmunity.

## Proteolipid protein

PLP is the most abundant protein in myelin and has two main isoforms: the full-length version which is almost exclusively expressed in the CNS, and the slightly shorter DM20 variant which is missing a loop of 35 amino acids and is only expressed in the periphery, thymus and lymph nodes (208, 209). The sequence excluded from the thymus-expressed DM20 variant contains the immunodominant PLP_139-151_ epitope which is a strong encephalitogen in some EAE models such as SJL/J (117, 210). SJL/J is a mouse model which is strongly predisposed to develop EAE with epitope spreading during subsequent relapses to other PLP epitopes and to MBP (117, 210). PLP_139-151_ is a frequent target of high avidity T cells in MS (45) – most likely due to its exclusion from thymic tolerance mechanisms – but several other encephalitogenic PLP peptides have been identified in humans such as PLP_104−117_, PLP_142−153_, PLP_184−199_, and PLP_190−209_, all of which can be presented by the MS risk allele HLA-DRB1*15:01 (47, 109). PLP is also the target of antibody responses, with up to 58% of pwMS in some studies showing antibody responses which are sensitive to protein conformation (114, 115).

Although fewer examples of CNS cross-reactivity between PLP and non-self antigens have been reported than for MBP, homology between human coronaviruses (HCoV) and PLP led to the isolation of several T cell clones from pwMS with dual-specificity for PLP and HCoV 229E and OC43 strains. Clonality and TCRVβ chain usage off cross-reactive T cell clones was confirmed, although the study did not enumerate frequency of these cells in peripheral blood of pwMS (119). The relevance of HCoV T cell molecular mimicry with CNS antigens is not certain and so far no further studies have replicated these findings with no large-scale sero-epidemiological studies have been presented. However, several reports globally of new MS cases and disease exacerbations following severe acute respiratory syndrome coronavirus 2 (SARS-CoV-2) infection or vaccination could suggest that exposure to SARS-CoV-2 antigens may trigger autoimmune attack on the CNS in some individuals (211), although there is currently no published functional evidence to support this. However, a recent *in silico* study showed that the nucleocapsid protein from SARS-CoV-2 shares significant overlap with several MS-associated myelin proteins including PLP (212), although this has not yet been investigated *in vitro*. On the other hand, increased numbers of MS cases following SARS-CoV-2 exposure could be attributed to bystander activation of pre-existing myelin-reactive T cells, or simply chance occurrences due to the immense number of people who were infected or vaccinated during the global Covid-19 pandemic. Further investigation is warranted to determine if cross-reactivity between coronavirus and neuronal antigens occurs *in vivo*.

As for MBP, sequence similarity between PLP and bacterial antigens have been reported, and one study demonstrated that EAE could be induced by both immunisation with PLP_139-151_ peptide or with homologous epitopes from *Haemophilus influenzae* and *Acanthamoeba castellanii* (81, 120, 121). The homologous peptide from *A. castellanii* was able to induce EAE in SJL/J mice and interestingly adoptive transfer of *A. castellanii*-specific T cells from female mice could also induce disease, however the same T cells derived from males could not (120). Further investigation of the PLP-homologous epitope from *H. influenzae* showed that induction of EAE required delivery of the pathogenic epitope in a recombinant Theiler’s encephalomyelitis virus (TMEV) vector, suggesting that CNS autoimmunity requires virus-specific activation of innate immune mechanisms in APCs such as Toll-like receptors (TLR) to fully break immune tolerance and lead to disease (81). In addition, further study of this model showed that mice infected with the recombinant *H. influenzae* TMEV had a T_H_1 CD4^+^ response to the homologous PLP_139-151_ peptide but no epitope spreading to PLP_178-191_. This was in contrast to the PLP_139-151_ TMEV, where epitope spreading to PLP_178-191_ could be detected and marked initial disease relapse in the SJL/J model (213). Amino acid substitution in the primary contact residue of PLP_139-151_ removed the ability of the virus to induce early EAE and therefore indicated that this residue was necessary for induction of pathogenic CD4^+^ T cell responses which drive early disease (121). Together these data indicate the strong adjuvant effect of viruses on autoreactive responses which are able to induce pathogenic T_H_1 CD4^+^ responses with rapid onset disease when combined with molecular mimics to myelin antigens. However, in this model epitope spreading readily occurred between PLP epitopes and mediated disease relapse and/or progression but this was not achieved between the foreign peptide and PLP. This suggests that further factors may be needed to sustain chronic CNS autoinflammation and long-term disease in this setting.

Although current evidence suggests a more important role for CD4^+^ T cells in MS pathogenesis, the high abundance of CD8^+^ T cells in MS lesions and oligoclonal TCR repertoires suggest that these expand and may be antigen-specific (58, 59). However, other studies have also shown myelin-specific CD8^+^ T cells to be present in peripheral blood at the same frequency in pwMS as in healthy individuals (214). The generally higher avidity of CD8^+^ TCR interactions with peptide:HLA and lower degeneracy of CD8^+^ T cells make cross-reactivity in this compartment less likely (215), features which perhaps emerged via evolution in order to limit cross-reactive responses with the ability to bind peptide:HLA class I complexes that are expressed almost ubiquitously on nucleated cells. However, examples of CD8^+^ T cell cross-reactivity do occur and Honma et al. described a HLA-A*03:01-restricted CD8^+^ T cell clone with specificity for PLP_45-53_ that could cross-recognise a peptide from *Saccharomyces cerevisae* in the context of HLA-A*02:01 (122). Although infrequent reports of myelin-reactive CD8^+^ T cell degeneracy may be in part due to the focus on CD4^+^ T cells in the MS research field. However, evidence from TCR sequencing of blood, CSF and MS lesions all suggest a clonal expansion of the CD8^+^ compartment in MS which may indicate migration of antigen-specific CD8^+^ T cells to the CNS during disease (58, 59), although the targets remain to be characterised and these could equally have a regulatory or suppressive phenotype. Interestingly, acute EBV infection – also known as infectious mononucleosis (IM) – is also characterised by enormously expanded oligoclonal CD8^+^ T cell repertoires directed against EBV antigens which subside over several weeks to months (216–218), however how these compare to the CD8^+^ compartment of MS patients remains to be determined.

Even though few examples exist of direct mimicry between PLP and foreign antigens, PLP remains one of the strongest encephalitogens, and *in vivo* inter- and intramolecular epitope spreading from initial PLP epitope responses is well-characterised in some EAE models as is described above (117, 210, 219). Further studies have shown EAE to be dependent on B cell presentation of PLP and MOG antigens to CD4^+^ T cells (220, 221), and efficacy of B cell depletion therapy in MS is thought to be partially due to removal of the antigen-presenting function of B cells. In contrast, fewer examples of epitope spreading from an initial PLP response have been reported for MS, however there are reports of spreading from MBP epitopes to PLP, and it is likely that each patient has a unique sequence of responses which develop through disease depending on their HLA type and other factors (222). However, this does not discount the possibility that an immunodominant T cell response to PLP_139-151_ in humans could, under the right circumstances, lead to an inflammatory event causing breakdown of the blood-brain barrier and lesion formation, after which epitope spreading to other CNS autoantigens may occur. These events are extremely difficult to investigate in humans due to the long prodromal phase of MS and also the difficulty and ethical barriers to sampling the affected tissue, ie. the brain and spinal cord.

## Myelin oligodendrocyte glycoprotein

MOG is a minor myelin component and is a transmembrane protein of the Ig superfamily. Overall, it constitutes less than 0.05% of myelin and is located in the outer membrane, and this location contributes to its relevance as an autoantigenic target as it is readily accessible by autoantibodies targeting its extracellular domain (223, 224). MOG was identified as a candidate autoantigen following observations that immunisation induces EAE and also the presence of MOG-specific autoantibodies and T cell responses in MS (45, 85–87, 94, 95, 98). In the EAE model, MOG-specific autoantibodies work synergistically with T cells to induce an inflammatory demyelinating disease which replicates observations from MS pathology (99, 225). An additional factor is MOG’s almost undetectable expression in the thymus (208), leading to escape of MOG-specific T cells from central tolerance mechanisms and which also partly accounts for the detection of MOG-specific T cells in some healthy controls as well as pwMS (85). MOG-reactive T cells retained in the immune repertoire may then become activated by environmental antigens with homology, which could lead to CNS inflammation and disease.

Mode and situation of cross-reactivity has been demonstrated to be important for priming of pathogenic cross-reactive responses, such as that demonstrated for cross-reactivity between MOG and the milk protein butyrophilin (BTN). BTN is a protein expressed in mammary tissue and is a major component of milk fat globules and has homology to MOG_76-87_ in its extracellular IgV-like domain. Immunisation of rats with MOG_76-87_ caused disseminated CNS inflammation characterised by infiltration of macrophages and CD4^+^ T cells, but interestingly this could be ameliorated by administration of the homologous BTN peptide either intravenously or intranasally with animals showing markedly decreased clinical scores (105). This amelioration is potentially due to induction of T_REG_ cells by the homologous BTN peptide, and this protective effect could in theory also occur in humans who consume bovine milk products into adulthood. However, further studies have shown that mechanisms of oral tolerance are poorly developed in babies, and tolerance may only be maintained when oral exposure is continued past a certain age – as has been demonstrated for oral tolerance to MBP in EAE (226). On the other hand, consumption of dairy products has also been linked to MS (227) and, although this finding remains controversial, cross-reactivity of antibodies which could bind homologous epitopes from both BTN and MOG were detected in the blood and CSF of pwMS (106). In addition to BTN, animals immunised with bovine casein were demonstrated to develop severe spinal cord pathology and demyelination which was attributed to induction of antibodies which bind to casein and cross-react with myelin associated glycoprotein (MAG) (154). The authors also noted increased antibody responses in pwMS compared to other neurological disease controls, suggesting that loss of tolerance to casein and molecular mimicry with MAG could contribute to disease in a subset of patients. However, the contribution of dietary milk proteins to MS pathogenesis remains to be fully elucidated in large cohort studies.

In addition to dietary antigens, sequence similarity has been identified between MOG and the human endogenous retrovirus W (HERV-W) envelope protein. This led to the finding that MOG autoantibodies could bind HERV-W protein (107) and one study used a nanotechnology approach to show a proof of principle that antibodies raised against MOG could cross-react with HERV-W (107). HERV are remnant genetic material left in the human genome following infection with retroviruses and constitute around 7% of the human genome (228). Initial investigation of HERV-W in MS brains was driven by isolation of HERV-W protein from sera, CSF and brain samples of affected individuals (229). It has also been demonstrated that HERV can also lead to activation of innate immune mechanisms through activation of Toll-like receptor 4 (TLR4) in APC and contributing to a proinflammatory milieu by driving T_H_1 responses. However, under these conditions, it is also possible that autoreactive cells could become activated via bystander mechanisms, and therefore the biological relevance of this MOG cross-reactivity with HERV-W remains to be verified in an MS cohort. Further studies by Sutkowski et al. demonstrated that the env protein of HERV-K18 – a superantigen capable of activating T cells expressing TCRVB13 – can become transcriptionally activated by EBV (230, 231). This activation of TCRBV13+ T cells was HLA-dependent but not restricted and may be involved in autoproliferative mechanisms in MS.

Although several factors likely contribute to epitope spreading as was discussed above for EAE, T cells isolated in response to the encephalitogenic epitope MOG_35-55_ have also been characterised as polyreactive to a similar sequence in neurofilament medium protein (NFM_15-35_) in mice (104). Both epitopes share the same TCR contact residues and could therefore potentially contribute to epitope spreading in disease. However, observations from EAE showed that NFM peptide did not expand MOG-specific T cells to a sufficient threshold to induce disease and NFM knockout mice had identical EAE disease to wild type animals indicating that, although NFM is immunogenic at the polyclonal level, it fails to expand high affinity MOG-specific T cells necessary for EAE induction (104).

## Guanosine diphosphate-L-fucose synthase, RAS guanyl releasing protein 2 and HLA-DR

Reactivity to guanosine diphosphate (GDP)-L-fucose synthase (GDPLFS) was first identified by systematically screening brain-infiltrating CD4^+^ T cell clones from MS patients for reactivity to a peptide library (70). T cell clones which responded to this antigen secreted T_H_2 cytokines and were identified in the CSF and brain of HLA-DRB3*02:02 positive individuals. Interestingly, GDPLFS clones were found to cross-recognise a number of bacterially-derived antigens as well as epitopes from other autoantigens such as MBP, PLP and MOG (70). T cell clones which reacted to GDPLFS could also respond to the dominant MBP_83-99_ epitope, with other clones showing reactivity to PLP_139-154_. Given the dual reactivity with bacterial epitopes, secretion of T_H_2 cytokines by the cross-reactive GDPLFS T cell clone is interesting as T_H_2 cells have a role in chronic inflammation and tissue repair (232). Given that the T cell clones respond to bacterial epitopes it is possible that they were initially primed in the gut. Also intriguing is that they were isolated from an individual with pattern II lesion pathology which is characterised by autoantibodies and complement deposition (233, 234), and an interesting avenue of future investigation would be to characterise whether the organ in which they were primed affects pathogenic T cell phenotype or subsequent lesion pathology in MS.

Auto-proliferation is defined as a spontaneous *in vitro* T cell proliferation without stimulus and is an observed feature of MS patient peripheral blood mononuclear cells (PBMC) in several studies (72, 235). Thorough examination of this phenomenon in pwMS was performed by Jelcic et al. who identified that there was a significant overlap of TCRVβ sequences from brain-infiltrating T cells in lesions and the auto-proliferating CD4^+^ T cell compartment in peripheral blood (72). T cell clones were isolated from the auto-proliferating blood compartment and their TCRVβ sequence was compared to those recovered from active lesions of the same HLA-DRB1*15:01 homozygous MS patient. The authors were then able to investigate specific clones which had infiltrated the brain and map their the antigen specificity using combinatorial peptide libraries. The cognate peptide from one T cell clone was mapped to a sequence from RAS guanyl-releasing protein 2 (RASGRP2), a previously unidentified MS autoantigen which is expressed in B cells, striatal neurons and cortical grey matter in the brain but is not a constituent of myelin, indicating that autoantigenic targets in MS do not necessarily need to be myelin proteins (72).

Identification of this situation in MS pathogenesis identifies a crucial link between peripheral activation of autoreactive CD4^+^ T cells by B cells and their subsequent infiltration into the CNS. In this scenario, B cells expressing high levels of HLA-DR present self-peptides from autoantigens such as RASGRP2 and stimulate auto-proliferative and auto-aggressive CD4^+^ T cells which subsequently enter the brain. After their infiltration into the CNS, these cells recognise RASGRP2 peptides presented in neuronal tissue and mediate inflammation. However, in this case the activating autoantigen in the periphery is the same as the target in the CNS, indicating that there should in theory be removal of autoreactive T cells via central tolerance mechanisms if the specified autoantigen is expressed in the thymus. RASGRP2’s expression in the thymus is not completely certain and it was not detected in thymic epithelial cells (71, 236). However, RASGRP2 expression was detected in some APCs in the thymus, indicating that RASGRP2-reactive T cells may be negatively selected from the repertoire via interaction with B cells and plasmacytoid dendritic cells (pDC) in the thymus (236).

Evidence that RASGRP2 autoreactivity could be stimulated by foreign antigens was demonstrated by Wang et al. who showed that peptides from MS associated pathogens EBV and *A. muciniphila* could also stimulate RASGRP2-specific CD4^+^ T cell clones *in vitro* (71). Interestingly, the cross-reactive EBV peptide sequences were derived from two lytic cycle antigens BHRF1 and BPLF1 which are expressed at high levels during acute infection where their functions are as a viral Bcl-2 homologue and a tegument deubiquitinase respectively (175, 237). No responses were observed to peptides from human HCMV or the bacterium *Prevotella histicola*, two pathogens have been negatively associated with MS (191, 206, 238). RASGRP2-specific T cell clones could also recognise HLA-derived self-peptides (HLA-SP) from HLA-DRB1 albeit with lower avidity, indicating that self-derived peptides are also partial agonists for CD4^+^ T cells targeting RASGRP2 and may help to maintain these T cells in the peripheral tissues via molecular mimicry (71). Some RASGRP2-specific T cell clones were shown to have an IFNγ^+^ T_H_1 phenotype, although the clone which was stimulated by EBV and *A. muciniphila* peptides had a T_H_2 phenotype and was isolated from a MS patient with type II lesion pathology (71, 72). *Ex vivo* analysis of T cell responses to peptides from RASGRP2, EBV and *A. muciniphila* showed these to be targets of responses in natalizumab-treated MS patients, indicating responses that had been primed *in vivo* and supporting their pathological relevance in this setting (71).

These data together suggest that HLA-DRB1*15:01-restricted RASGRP2-specific T cells can become activated in the periphery via both self-antigens and foreign peptides derived from EBV and *A. muciniphila*, and also that these CD4^+^ T cells specifically infiltrate the brain where they can be found in active lesions. This supports the hypothesis in MS that CNS autoimmunity is either initiated or maintained by pathogenic CD4^+^ T cell responses which are initially primed by exogenous antigens but can respond to autoantigens, leading to their recruitment to the brain where they mediate inflammatory tissue damage. These data are striking, and further investigation of RASGRP2 in large MS cohorts to establish the frequency of responses to this autoantigen are warranted.

## Conclusion

It is clear that cross reactivity between a variety of microbial agents and host CNS autoantigens has been demonstrated, though in most cases of unclear relevance for MS pathogenesis due to experiments with low numbers of T cell clones or in artificial experimental animal models. However, in large sero-epidemiological studies, EBV – and to some extent HHV-6A – stand out. Especially for EBV, clear evidence for molecular mimicry epitopes has been demonstrated and antibody responses directed against these epitopes strikingly associate to an increased risk for MS. This strongly supports their role in MS pathogenesis, perhaps as markers for a concomitant T cell autoimmunity.

These observations make a case for therapeutic intervention, in particular if EBV drives the chronicity of the disease, perhaps with EBV-specific low molecular antiviral agents, or vaccination of individuals at risk for MS or at onset of disease. However, due to the multiple CNS autoantigen mimics now identified in EBNA1_390-440_, development of future EBV vaccines or adoptive T cell therapies which include this antigen should either be designed with extreme caution or avoided altogether lest MS disease be triggered or exacerbated. The MS research field has reached an exciting stage, however further extensive research is needed to fully elucidate the role of foreign antigens which mimic autoantigens in the development and progression of CNS demyelinating disease.

## Author contributions

OT: Conceptualization, Writing – original draft, Writing – review & editing. TO: Funding acquisition, Supervision, Writing – review & editing.
